# Pacing interventions in non-responders to cardiac resynchronization therapy

**DOI:** 10.3389/fphys.2023.1054095

**Published:** 2023-01-26

**Authors:** Nadeev Wijesuriya, Mark K. Elliott, Vishal Mehta, Felicity De Vere, Marina Strocchi, Jonathan M. Behar, Steven A. Niederer, Christopher A. Rinaldi

**Affiliations:** ^1^ School of Biomedical Engineering and Imaging Sciences, King’s College London, London, United Kingdom; ^2^ Department of Cardiology, Guy’s and St Thomas’ NHS Foundation Trust, London, United Kingdom

**Keywords:** CRT, CRT non-response, endocardial pacing, leadless cardiac resynchronization therapy, conduction system pacing

## Abstract

Non-responders to Cardiac Resynchronization Therapy (CRT) represent a high-risk, and difficult to treat population of heart failure patients. Studies have shown that these patients have a lower quality of life and reduced life expectancy compared to those who respond to CRT. Whilst the first-line treatment for dyssynchronous heart failure is “conventional” biventricular epicardial CRT, a range of novel pacing interventions have emerged as potential alternatives. This has raised the question whether these new treatments may be useful as a second-line pacing intervention for treating non-responders, or indeed, whether some patients may benefit from these as a first-line option. In this review, we will examine the current evidence for four pacing interventions in the context of treatment of conventional CRT non-responders: CRT optimization; multisite left ventricular pacing; left ventricular endocardial pacing and conduction system pacing.

## Introduction

Cardiac Resynchronization Therapy (CRT), in addition to optimal medical therapy, is a widespread and successful treatment for patients with dyssynchronous heart failure (HF) (Glikson et al., 2021). Conventionally, CRT involves transvenous systems delivering biventricular (BiV) pacing from leads in the right ventricle (RV), and a cardiac vein via the coronary sinus (CS) to achieve epicardial left ventricular (LV) stimulation. There is strong evidence that CRT improves HF symptoms whilst reducing HF hospitalisations and improving mortality in indicated patients (McAlister et al., 2007).

Unfortunately, there is a subgroup of high-risk patients who have a poor therapeutic response to CRT, so-called “CRT non-responders” representing between 30% and 50% of CRT patients (Young et al., 2003). Several factors have been proposed to contribute to this limited efficacy. Cardiac venous anatomy significantly restricts the pacing location of the LV lead, which may lead to difficulty in targeting optimal sites, and avoiding areas of transmural scar (Wouters et al., 2021). LV scar is present in up to 40% of CRT candidates, and predicts poor response (BLEEKER et al., 2006; Chalil et al., 2007; Leyva et al., 2011; Wong et al., 2013). In addition, modelling studies demonstrate that conventional CRT does not replicate physiological activation across the endocardium and in some instances may be pro-arrhythmic (Mendonca Costa et al., 2019).

Treatment of CRT non-responders is extremely difficult, and this cohort of patients is known to have poor outcomes (Vijayaraman et al., 2022a). In recent years, several novel pacing interventions have been investigated to assess whether these therapies can provide benefit when clinical improvement does not occur despite BiV or when conventional transvenous implantation was not successfully achieved. These interventions include: optimisation of atrioventricular (AV) and interventricular (VV) delays; multisite LV pacing; LV endocardial pacing and conduction system pacing (CSP).

In this review, we will examine the current data for these four pacing interventions in the treatment of CRT non-responders, discuss the limitations of the current body of evidence, and provide opinions on future directions in this field.

### Optimisation of atrioventricular (AV) and interventricular (VV) delays

Delay optimisation has been the subject of investigation since the advent of CRT, arising from the theory that optimisation of both passive and active filling will maximise cardiac output, thereby improving outcomes. Observational studies have reported acute haemodynamic and electrical benefits of AV/VV optimisation in patients receiving CRT (Jansen et al., 2006; AlTurki et al., 2019), however, clinical trials have not consistently reported long term benefits (Brabham and Gold, 2013). The SMART-AV (Ellenbogen et al., 2010) trial which randomised 980 patients in a 1:1:1 ratio to CRT with an empirical AV delay of 120 ms, echocardiographically optimised AV delay, or AV delay optimised with SmartDelay, an electrogram-based algorithm. This study demonstrated no significant improvements in either AV optimisation arm over empirical settings based on LV end systolic volume improvement or clinical improvement at 6 months.

There are several reasons why acute mechanistic data examining haemodynamic benefits of AV delay optimisation do not translate to improve outcomes in clinical trials. The intrinsic PR interval is variable, especially in response to factors such as autonomic tone and exercise (Lee et al., 1995). As such, the optimal AV delay programmed in clinic may not reflect the patient’s real-world physiology.

Another reason that AV optimisation has not shown significant positive results may be because the majority of patients respond very well to empirical BiV pacing. Thus, the beneficial effect will likely be small in an unselected CRT population. There may however be a role for optimisation in a selected CRT non-responder group. Brown et al. reported that in 32 echocardiographic CRT non-responders, CRT optimization significantly improved LV ejection fraction from 31.8% ± 4.7% to 36.3% ± 5.9% (*p* < .001) and LV end-systolic volume from 108.5 ± 37.6 to 98.0 ± 37.5 mL (*p* = .009). Additionally, speckle-tracking measures of LV strain significantly improved by 2.4% ± 4.5% (transverse; *p* = .002) and 1.0% ± 2.6% (longitudinal; *p* = .017) and aortic to pulmonic valve opening time, a measure of interventricular dyssynchrony, significantly (*p* = .040) decreased by 14.9 ± 39.4 ms (Brown et al., 2022). Similar conclusions were reached by Naqvi et al., who reported improved echo-derived strain measures of dyssynchrony in a series of 8 clinical non-responders receiving AV and VV optimisation (Naqvi et al., 2006). Whilst these results appear promising, they have not been consistently replicated. Another small study, in 8 patients classified as both echocardiographic and clinical non-responders, reported no improvements in echo outcomes after receiving CRT optimisation (Sepši et al., 2013). Larger randomised studies specifically targeting a non-responder population are needed to provide more definitive answers to this potentially practice changing intervention.

### Multi-point and multi-lead pacing

Multi-point pacing (MPP) and multi-lead pacing, such as “triventricular” (TriV) pacing is a well-studied area in the field of CRT non-response. Pacing the LV from multiple locations is an attractive concept as it potentially addresses the problems caused by ischaemic scar or other areas of slow conduction velocity that reduce the efficacy of CRT by affecting the LV paced wavefront. Several studies testing the efficacy of these interventions have been performed in non-responder populations. In the SMART-MSP trial, 102 patients who had an unchanged or worsened clinical composite score, (composed of all-cause mortality; HF events; patient global assessment; and NYHA HF classification) at 6 months post-CRT had LV MPP turned on (Saba et al., 2022). They found that 51% of these patients became clinical responders at 12 months follow up, and concluded that LV MPP is beneficial in the treatment of non-responders. However, this trial did not include any echocardiographic data, as such the primary endpoint was a subjective measure. Furthermore, a criticism of this study was the lack of a control group, in particular, that a significant proportion of CRT non-responders at 6 months may have become responders at 12 months even in the absence of MPP. Indeed, this was demonstrated in Phase 1 of the MORE-CRT trial (Leclercq et al., 2019), which randomised 467 non-responders at 6 months post CRT to MPP-ON or MPP-OFF. This trial reported no significant difference in echo response between the groups at 12 months follow-up as evaluated in a blinded echo core lab. In both the MPP-ON (31.8%) and MPP-OFF (33.8%) groups a subset of non-responders converted into responders at follow-up. The authors suggested that there may be a delayed response to biventricular pacing beyond the initial 6 months owing to a myocardial substrate that needs more time to fully undergo reverse remodelling, or heart failure medication that continues to be up titrated-whether that be with MPP, MSP, or conventional CRT. A recently published meta-analysis by Mehta et al. reported that in randomised studies, there is no difference between MPP and conventional CRT (Mehta et al., 2021).

**FIGURE 1:**
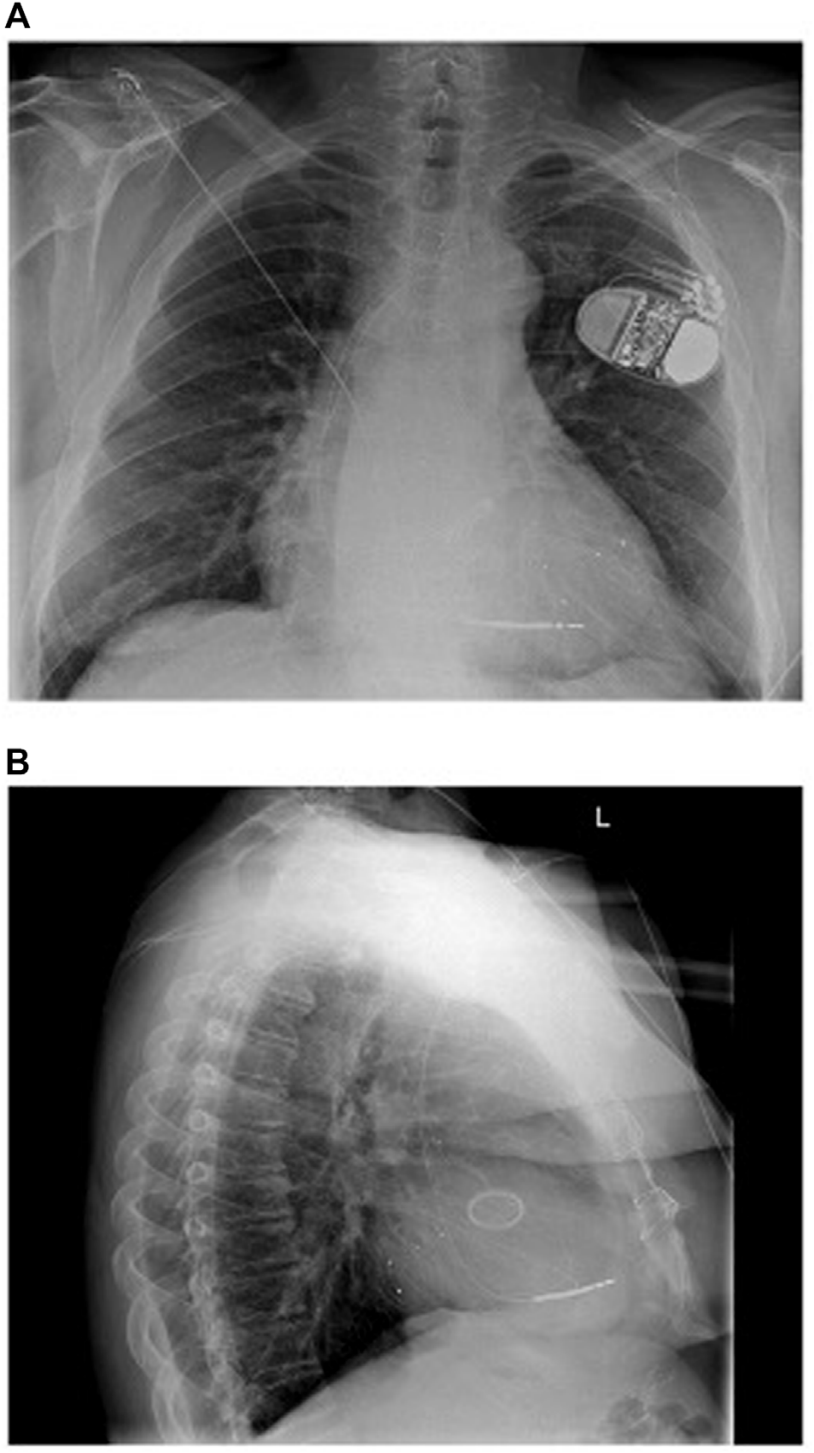
**(A)** Anteroposterior and **(B)** Lateral chest radiograph views 1 day post implant of triventricular CRT system with one LV lead in posterolateral vein and another in a lateral vein. Reproduced from reference 23, Gould et al., with permission.

Multi-lead pacing, that is, the placement of an additional lead, most commonly in the LV to provide Triventricular (TriV) pacing (Figure 1), has also been evaluated in randomised control trials. The V3 trial (Bordachar et al., 2018) and STRIVE-CRT (Gould et al., 2022) are important negative trials which showed no significant difference in clinical or echocardiographic outcomes between standard of care and multi-lead pacing in unselected CRT populations. A meta-analysis of 415 patients by Elliott et al. (Elliott et al., 2022a) again reported no difference between TriV pacing and conventional BiV pacing.

Acute haemodynamic studies in both animals (Ploux et al., 2014; Heckman et al., 2020) and humans (Sohal et al., 2015) have reported that LV multi-lead pacing may provide acute haemodynamic benefits over BiV CRT in subjects who are “acute haemodynamic non-responders” to conventional CRT. These studies have yet to be replicated on a larger scale with robust outcome data. Until such time, it cannot be extrapolated that there is a significant benefit of implanting an additional LV lead in non-responders.

### Left ventricular endocardial pacing

Endocardial pacing provides more physiological activation than epicardial pacing (Bordachar et al., 2012), and importantly, gives the benefit of unrestricted LV pacing locations, which can be vital in patients with factors including ischaemic scar or lack of suitable cardiac venous targets, through unfavourable anatomical characteristics such as difficult coronary sinus os access, a persistent left sided subclavian vein, or tributaries which are too small to support a lead. The emergence of LV endocardial pacing as a potential treatment for conventional CRT non-responders has been driven primarily by mechanistic studies which have consistently reported acute haemodynamic benefits for endocardial pacing versus conventional CRT (Derval et al., 2010; Ginks et al., 2012; Shetty et al., 2014; Sohal et al., 2014; Behar et al., 2016). The optimal LV pacing locations reported were highly variable, but in these studies, the endocardial site with the largest improvement in acute haemodynamic response (AHR) was consistently superior to conventional BiV pacing. Behar et al. (Behar et al., 2016) reported from a total of 135 sites tested in 8 patients that AHR was significantly greater when temporary pacing the same myocardial segment endocardially versus epicardially (15.2 ± 10.7% vs. 7.6 ± 6.3%; *p* = 0.014) and resulted in a shorter paced QRS duration (137 ± 22 ms vs. 166 ± 30 ms; *p* < 0.001). Interestingly, Sohal et al. (Sohal et al., 2014) reported an acute haemodynamic study of 10 patients with biventricular CRT devices. The optimal LV endocardial pacing site was at the same location as the existing epicardial LV lead in only four patients. An acute haemodynamic study performed by Padeletti et al. in 11 subjects also demonstrated that the optimal LV endocardial site in each patient significantly improved LV performance compared to conventional epicardial LV stimulation (Padeletti et al., 2012).

Mechanistic studies have also provided insight into which patients may benefit from endocardial pacing rather than epicardial LV pacing. Ginks et al. (Ginks et al., 2012) performed electroanatomical mapping to determine the intrinsic LV activation pattern and a haemodynamic study in 10 patients with LBBB referred for CRT. The authors reported that the majority (71%) of patients with non-ischemic heart failure and a line of conduction block causing LBBB responded to conventional CRT. In contrast, those with myocardial scar, and the absence of a line of conduction block, i.e. where LBBB was caused by homogenously slow conduction from the LV septum to the lateral wall, often required endocardial or multisite pacing to achieve CRT response. Non-responders have also specifically been studied in this setting. Gelder et al. (van Gelder et al., 2016) performed an acute haemodynamic study in 24 clinical CRT non-responders. They found that the initially implanted system generated an AHR ≥15% in five patients after A-V and V-V optimisation. Among these 5, three with posterolateral transvenous epicardial leads had no significant AHR increase with LV endocardial pacing. One of the two other patients with transvenous apical epicardial leads had an AHR rise from 19.7% to 66% with LV endocardial pacing. Nine of the 19 remaining patients had an increase in AHR to ≥15% at the optimal endocardial LV pacing position.

Initial systems delivering permanent LV endocardial pacing were lead-based. ALSYNC (Morgan et al., 2016) was a prospective clinical investigation of 118 patients who received a trans-septal (inter-atrial) LV endocardial pacing lead. Ninety patients (76.2%) had a failed epicardial lead or suboptimal cardiac venous anatomy and 28 (23.8%) were non-responders to previous CRT. At 6 months, the New York Heart Association (NYHA) class improved in 59% of patients, and 55% had LV end-systolic volume (LVESV) reduction of 15% or greater. Those patients enrolled after CRT non-response showed similar improvement, with 47% of patients having an improvement in LVESV of ≥15%, and 5% having an improvement ≥30%. Unfortunately in this study, 14 transient ischaemic attacks (9 patients, 6.8%) and five non-disabling strokes (5 patients, 3.8%) were observed. This prohibitively high embolic risk and the requirement for lifelong anticoagulation has motivated the development of novel leadless LV endocardial pacing systems.

Delivering CRT via leadless LV endocardial pacing has several potential advantages compared to lead-based systems. Complete device endothelialisation reduces the stroke risk and anticoagulation requirement (Echt et al., 2010), and devices can be implanted in patients where venous access or infection issues preclude both conventional and lead-based endocardial CRT. (Gamble et al., 2016). In addition, leadless pacing can avoid the numerous long-term complications associated with transvenous leads including: insulation breach, fracture (1%–4%); venous obstruction (8%–21%); and infection (1%–2%) (Bernard, 2016) which often result in the need for high risk extraction procedures.

**FIGURE 2:**
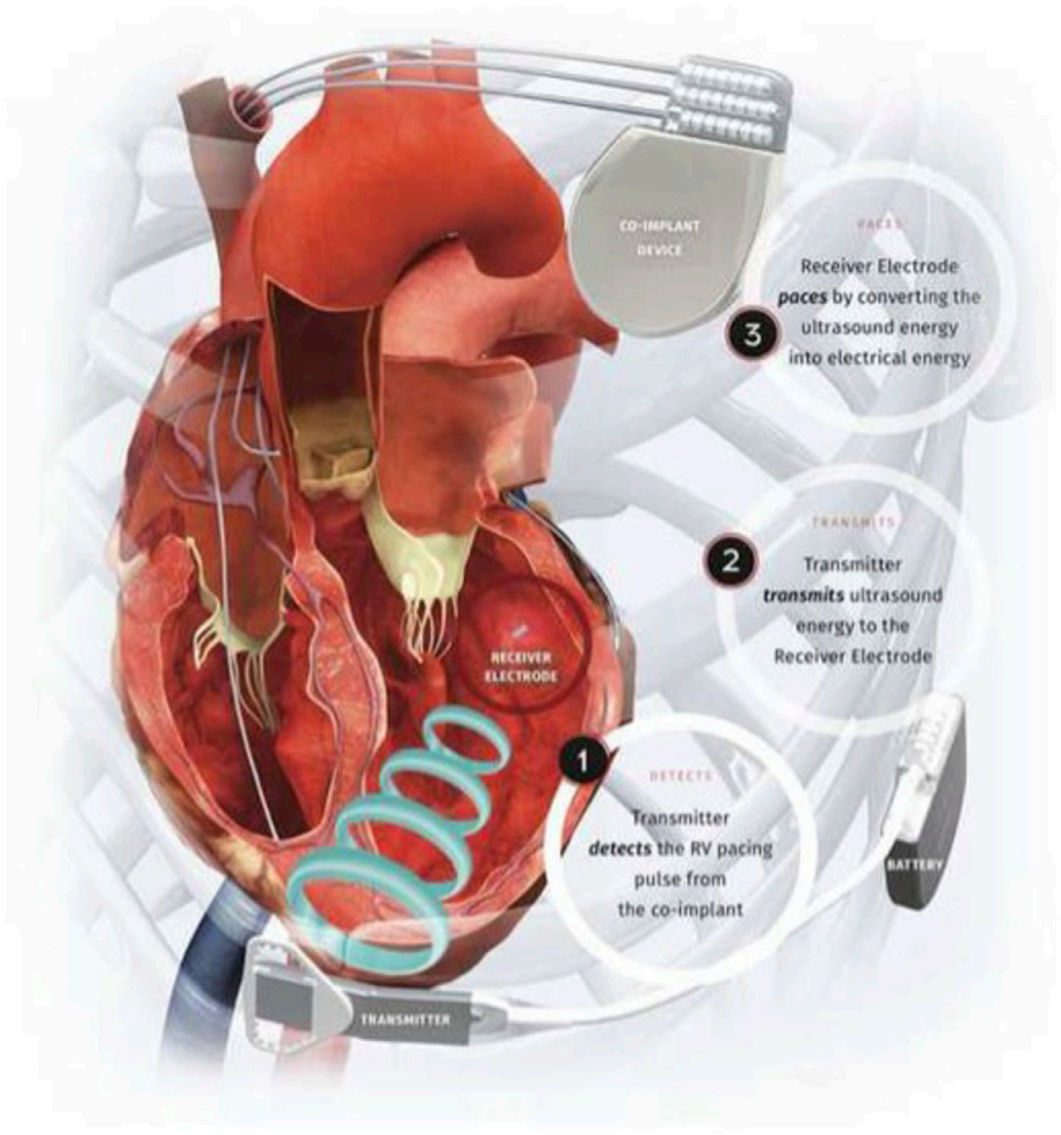
Components of the WiSE-CRT System. Reproduced from reference 64, Elliott et al., with permission.

The WiSE-CRT system (EBR Systems Inc., Sunnyvale CA) is the only commercially available leadless LV pacing system (Auricchio et al., 2014). The system consists of a battery connected to an ultrasound transmitter, which is implanted subcutaneously at the 4th, 5th, or 6th intercostal place, and the receiver electrode, which is implanted in the LV cavity via aortic or trans-septal access (Figure 2). The system requires the patient to have a “co-implant” *in situ* capable of producing continuous RV pacing, which can be either a conventional device, such as a pacemaker or implantable cardiac defibrillator (ICD), or a leadless pacemaker such as MICRA™ (Medtronic, Minneapolis MN). The transmitter and battery detect an RV pacing pulse emitted by the co-implant. Within 10 ms of detection of the RV pacing spike, the transmitter emits a number of ultrasound pulses to locate the receiver electrode. Once the transmitter is electronically optimally aligned, a longer ultrasound wave is emitted, which is detected and converted to a pacing stimulus by the receiver electrode. This results in LV pacing, and thereby BiV pacing.

Several observational studies have demonstrated that treatment with WiSE-CRT can deliver echocardiographic CRT response (Auricchio et al., 2014; Reddy et al., 2017; Sieniewicz et al., 2020; Okabe et al., 2022). A recent meta-analysis of these studies (Wijesuriya et al., 2022a) reported that in a total of 181 patients, there was a mean increase in LVEF of 6.3% (Mean difference 6.3, 95% Confidence Interval (4.35, 8.19) *p* < 0.001, with low heterogeneity (*p* = 0.84, I^2^ < 0.001%). The echocardiographic response rate (variably defined between studies as either a reduction in LVESV of >15%, an improvement in LVEF>5%, or an improvement in LVEF>10%) was 54% in a population where 22% were non-responders to conventional CRT.

A sub-analysis of the non-responder population of the WiSE-CRT registry was performed by Sidhu et al. (2020) The authors reported that in 18 patients, endocardial pacing resulted in a significant reduction in QRS duration compared with intrinsic QRS duration (26.6 ± 24.4 ms; *p =* .002) and improvement in left ventricular ejection fraction (LVEF) (4.7 ± 7.9%; *p* = .021). Overall, 55.6% of patients had improvement in their clinical composite score (consisting of number of hospitalizations with decompensated heart failure; survival to follow-up; improvement of ≥1 NYHA functional class; or improvement in their global assessment) and 66.7% had a reduction in LVESV ≥15% and/or absolute improvement in LVEF ≥5%. These results, albeit in a small patient cohort, provide preliminary favourable feasibility data of WiSE-CRT in treatment of non-responders. The ongoing SOLVE-CRT trial (NCT02922036) (Singh et al., 2021), a multicentre interventional study, will provide further valuable information about the efficacy of this new treatment modality.

### Conduction system pacing

Conduction system pacing (CSP) is an area of rapidly growing interest, built upon the attractive concept of restoring completely physiological ventricular activation. Initial studies in lead-based CSP focused on His Bundle Pacing (HBP). HBP achieves excellent cardiac resynchronization, but implantation can be difficult with success rates varying from 56%–95% (Bhatt et al., 2018; Sharma et al., 2018; Vijayaraman et al., 2018). Concerns about ventricular under-sensing and rising thresholds have emerged during long-term follow up (Lustgarten et al., 2019; Zanon et al., 2019). Left Bundle Branch Area Pacing (LBBAP) is a novel form of CSP which involves screwing a pacing lead deep into the interventricular septum from the RV in order to capture the left bundle system (Huang et al., 2019; Zhang et al., 2019). This technique has produced encouraging results from observational studies, with reported success rates of 80%–94%, (Padala and Ellenbogen, 2020), and significant improvements in LV systolic function (Zhang et al., 2019). Robust data from randomised control trials, however, is currently lacking. Current evidence, especially with regards to the role of CSP in non-response, is limited to *in silico* studies and observational studies.

**FIGURE 3:**
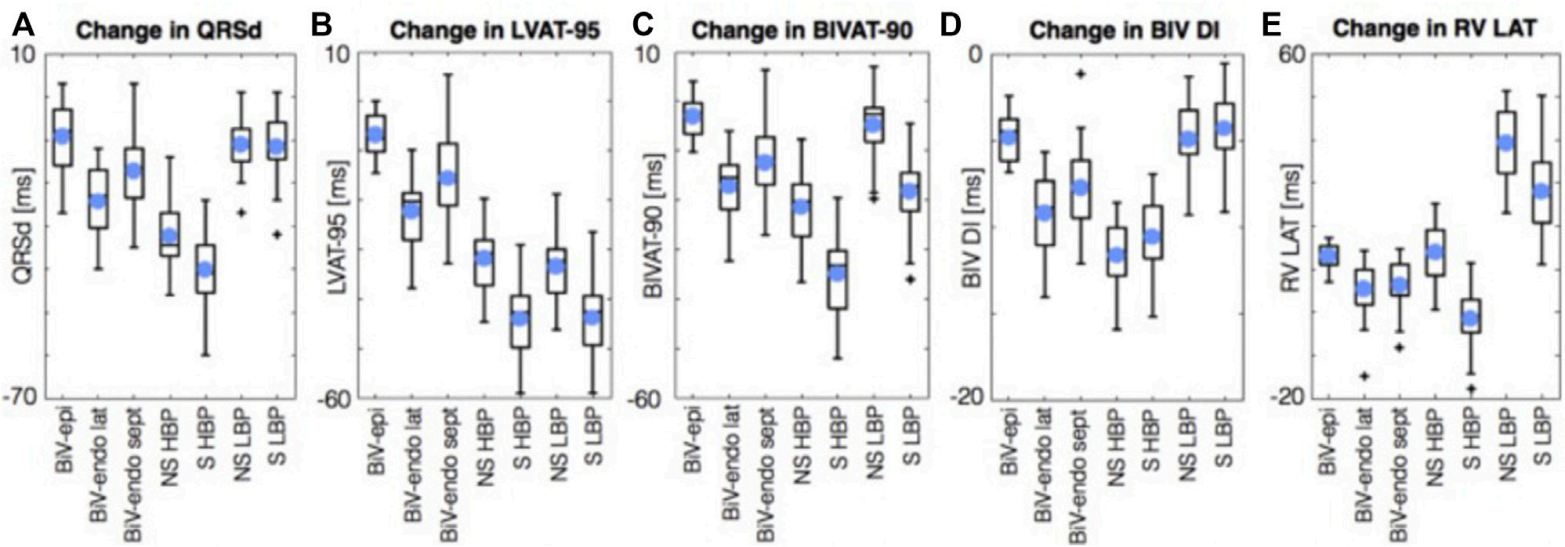
Simulations results using 24 four chamber heart meshes. Boxplots of the change in QRSd, **(A)** LVAT-95, **(B)** BIVAT-90, **(C)** BIV DI **(D)**, and RV LAT **(E)** from baseline with BiV-epi pacing at the optimal location, BiV-endo lateral pacing at the optimal location, BiV-endo septal pacing (BiV-endo sept), S- and NS-HBP, and S- and NS-LBP. *Light blue circles* represent mean values. *Plus symbols* represent outliers. BiV, biventricular; BIV DI, biventricular dyssynchronous index; BIVAT-90, 90% biventricular activation time; endo, endocardial; epi, epicardial; HBP, His-bundle pacing; LAT, lateral; LBP, left bundle pacing; LV, left ventricle; LVAT-95, 95% left ventricular activation time; NS, non-selective; QRSd, QRS duration; RV LAT, right ventricular latest activation time; S, selective; sept, septal. Reproduced from reference 55, Strocchi et al., with permission.

Strocchi et al. (2020) performed an *in silico* study examining ventricular activation times on 24 four chamber heart meshes in the presence of simulated left bundle branch block (LBBB). They simulated BiV epicardial and BiV endocardial pacing, as well as HBP and LBBAP. They reported that HBP was superior (*p* < .05) to BiV endocardial and conventional BiV pacing with regards to reduction in LV activation time (AT) and interventricular dyssynchrony, (Figure 3). LBBAP reduced LV activation times but not interventricular dyssynchrony compared to conventional CRT and BiV endocardial pacing, due to late RV activation. The RV latest AT was higher with LBP than with HBP (141.3 ± 10.0 ms vs. 111.8 ± 10.4 ms). Optimizing AV delay during LBP reduced RV latest AT (104.7 ± 8.7 ms) and led to comparable response to HBP. These results suggest that CSP provides an electrical benefit over conventional CRT in unselected LBBB patients. We may extrapolate from this that CSP might be beneficial in patients who have not responded to conventional CRT.

Data from observational trials comparing conventional CRT with CSP in both unselected patients and non-responders has had variable outcomes. Non-randomised observational studies by Chen et al. and Vijayaraman et al. (Vijayaraman et al., 2022b; Chen et al., 2022) demonstrated improvements in QRS duration and echocardiographic outcomes with CSP compared to conventional BiV pacing in *de novo* implant patients, but this has not been consistently replicated (Upadhyay et al., 2019a; Toding Labi et al., 2022). Interestingly, Vijayaraman et al. performed a further observational study of 200 patients who underwent LBBAP for either inability to place a transvenous LV epicardial lead (Group 1, *n* = 156), or CRT non-response (Group 2, *n* = 44) (Vijayaraman et al., 2022a). QRS duration, LVEF and NYHA class improved in both groups, but more so in group 1. At mean 12 months follow-up the primary endpoint of death or HF hospitalisations was significantly lower in group 1 than group 2 (13% vs. 30%; HR 0.357; *p* = .007). The incidence of clinical and echocardiographic improvements in Group 1 was similar to those observed in patients undergoing conventional CRT in clinical trials. The authors concluded that LBBAP is a reasonable alternative to BiV CRT but more work is needed to assess its efficacy in non-responders.

An emerging field is the potential improvement in electrical synchronisation obtained through optimising conventional CRT with sequential CSP-LV pacing, known as His-Optimised CRT (HOT-CRT) or LBP-Optimised CRT (LOT-CRT). A mechanistic study of 11 patients showed that pressure-volume derived stroke volume was optimal when LV pacing was combined with HBP, suggesting that sequential CSP-LV activation provides benefit by preserving intrinsic RV activation (Padeletti et al., 2016). A 25 patient feasibility study of HOT-CRT (Vijayaraman et al., 2019) demonstrated that QRS duration at baseline was 183 ± 27 ms and significantly narrowed to 162 ± 17 ms with biventricular pacing (*p* = 0.003), to 151 ± 24 ms during HBP (*p* < 0.0001), and further to 120 ± 16 ms during HOT-CRT (*p* < 0.0001). During a mean follow-up of 14 ± 10 months, LV ejection fraction improved from 24 ± 7% to 38 ± 10% (*p* < 0.0001), and NYHA functional class changed from 3.3 to 2.04. Twenty-one of 25 patients (84%) were clinical responders while 23 of 25 (92%) demonstrated an echocardiographic response. A LOT-CRT feasibility study (Jastrzębski et al., 2022) reported a clinical response rate of 76% in 91 patients. Although performed in small cohorts with no control groups, the high CRT response rates seen in these studies raise the question of whether addition of CSP to BiV pacing in CRT non-responders will be efficacious in a significant number of patients. Larger studies of non-responder patients will of course be needed in this regard, and it will be important to consider safety outcomes as well as heart failure outcomes, given both the additional infection risk associated with upgrade procedures, as well as long-term risks of lead-lead interaction, thrombosis and tricuspid regurgitation which increase with implantation of additional transvenous leads (Bernard, 2016). This is particularly pertinent as while CSP is becoming more widespread worldwide, (Performance Reports | Medtronic CRHF, 2022), extraction of CSP leads remains a low-volume procedure with a very small evidence base (Wijesuriya et al., 2022b).

Recent advances in WiSE-CRT implantation have brought about the ability to perform CSP via a leadless LV endocardial approach, potentially circumventing long-term lead related issues (Elliott et al., 2021; Elliott et al., 2022b; Wijesuriya et al., 2022c). The endocardial receiver electrode component of the WiSE-CRT system has traditionally been implanted at the LV lateral wall using a retrograde femoral arterial approach, however the emergence of a trans-septal implant technique gives the operator the ability to find a stable delivery sheath position on the LV septal wall using deflectable sheaths such as the FlexCath (Medtronic, Minneapolis MN). Initially the LV septum is mapped using a decapolar catheter, enabling the electrode to be targeted to the site of a pre-systolic potential, with the aim of capturing the His-Purkinje system. In a case series of 8 patients, the implant success rate was 100% (Elliott et al., 2022b), with a significant reduction in QRS duration (187.1 ± 33.8 ms vs. 149.5 ± 15.7 ms; *p* = .009). One of these 8 patients was a CRT non-responder, with the remainder being transvenous LV epicardial lead failures. Further data in this regard will come with time-LV septal implants are projected to increase in view of an improved safety profile of trans-septal compared to large-bore aortic access, and historically, around 1 in 5 patients receiving WiSE-CRT have been conventional CRT non-responders (Wijesuriya et al., 2022a). Much work is required before this are progresses towards randomised trials-in the first instance electroanatomical mapping data determining the ventricular activation pattern of a WiSE-CRT septal implant will shed light on questions such as whether His-Purkinje tissue is captured, and whether this is electrically superior to LV endocardial pacing from alternative sites.

## Discussion

We now have several new and/or emerging CRT options which all theoretically have the potential to treat non-responders to conventional BiV epicardial CRT (Table 1). Whilst there have been some positive outcomes reported from observational studies, these have not been consistently replicated in larger trials. We believe that there are several reasons for these inconsistencies.

**TABLE 1:** Summary of different pacing options for CRT non-responders.

Pacing option	Evidence summary for CRT non-responders
AV/VV delay optimisation	Small observational studies—conflicting data Jansen et al., 2006; Ellenbogen et al., 2010; Brabham and Gold, (2013); AlTurki et al., 2019
Multi-point pacing (MPP)	MORE-CRT RCT—no benefit in MPP Leclercq et al., 2019
Multi-site pacing (MSP)	Mechanistic studies—Acute haemodynamic benefit in of MSP in acute haemodynamic non-responders to conventional CRT. Ploux et al., 2014; Sohal et al., 2015; Heckman et al., 2020 No larger studies as yet.
LV endocardial pacing	Small observational studies—Lead-based and leadless endocardial pacing may achieve echocardiographic and clinical response in a significant proportion of non-responders. (van Gelder et al., 2016; Sidhu et al., 2020 SOLVE-CRT study ongoing
Conduction system pacing	Observational studies—HBP, LBP, HOT-CRT and LOT-CRT may give potential improvements in electrical resynchronisation obtained by preserving intrinsic RV activation, yet to be demonstrated in a non-responder population. Vijayaraman et al., 2019; Vijayaraman et al., 2022b; Jastrzębski et al., 2022; Toding Labi et al., 2022

The foremost issue is that conventional biventricular CRT is an excellent treatment option for HF. In appropriately selected candidates, response rates are 60%–70% (Young et al., 2003). Observational studies tend to report at most a fairly mild improvement in indices such as AHR^29 44^ in head-to-head comparisons between novel CRT therapies and conventional CRT in *de novo* patients. Because of relatively small projected impact on measurable parameters, it will always be very difficult for the novel therapies to demonstrate superiority compared to conventional CRT in an unselected group of patients. In particular, there is generally attenuation of effect size in larger clinical trials compared to observational studies. In small single-centre trials, there may be a degree of recruitment bias, possibly by avoiding subjects with unfavourable CRT characteristics such as atrial fibrillation or right bundle branch block. The influence of bias is less likely to be prominent in larger multicentre studies. As such, therapies which initially sound promising, such as AV/VV optimisation (Brabham and Gold, 2013) and multipoint/multi-lead pacing (Elliott et al., 2022a) have lost momentum due to negative results in clinical trials of unselected patients. In actuality, their primary benefit could have been demonstrated by specifically targeting a non-responder population, where the potentially larger impact on measurable parameters may be adequate to power randomised trials at a reasonable sample size.

**FIGURE 4:**
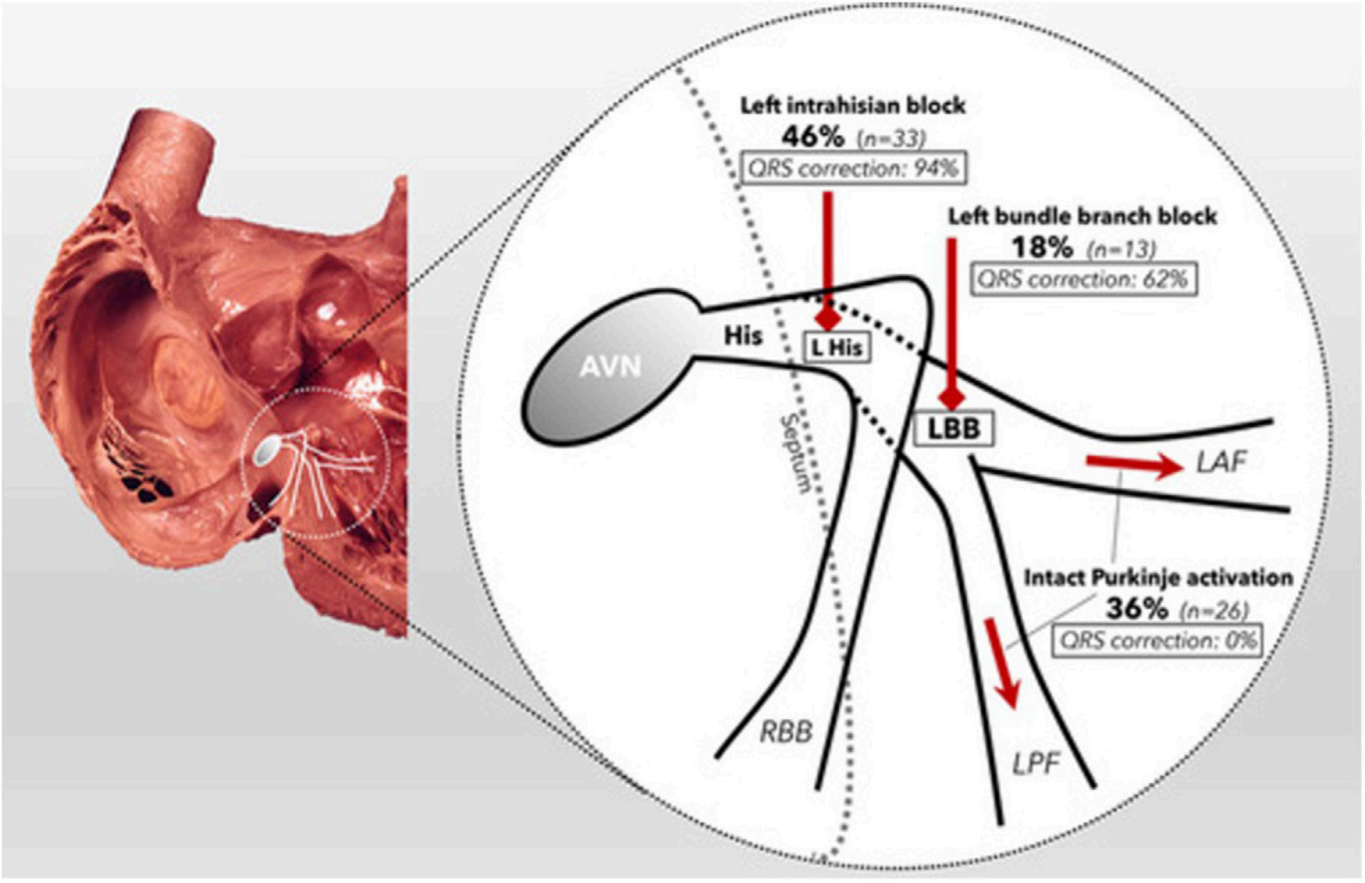
Schematic of the proximal conduction system, demonstrating the prevalence of each form of conduction disorder within the cohort (bold) and the percentage of patients whose QRSd was corrected by HBP (italic). Reproduced from reference 68, Upadhyay et al., with permission.

This brings us to the point of patient selection. Mechanistic studies have generally shown that there is significant variability in the optimal pacing site between individuals (Shetty et al., 2014; Sohal et al., 2014; Behar et al., 2016). This may be due to factors such as scar location, phenotype of conduction disturbance and aetiology of heart failure. (Ginks et al., 2012) For example, Upadhyay et al. (Upadhyay et al., 2019b) demonstrated in a temporary pacing and electroanatomical mapping study of 72 subjects with LBBB that whilst CSP overcame proximal block in 64% of patients, 36% of their cohort displayed “intact Purkinje activation” where conduction disturbance is caused by more distal diffuse disease (Figure 4). In these patients, the QRS duration was not corrected by HBP.

Lastly, CRT non-responders are a highly heterogenous group of patients where failure of conventional CRT may occur for a number of reasons. There may be an optimal pacing site for each patient, but currently, our pre-assessment procedures do not aim to identify this as part of standard clinical practice. Whilst prediction of optimal pacing sites has been demonstrated in a research setting (Duckett et al., 2011; Sieniewicz et al., 2018; Sohal et al., 2021) making this operational in a non-invasive, cost-effective manner will be more difficult. Further work involving pre-procedural imaging such as MRI and CT to define scar may yield positive results in this regard moving forward. Until such time as this personalised treatment can be delivered, it seems unlikely that any one alternative pacing modality will demonstrate superiority over a successful treatment like conventional CRT in larger clinical trials.

An additional issue in this field is difficulty in interpreting the current evidence base due to the lack of a standardised definition of CRT response. The most widely accepted definition involves an assessment of left ventricular reverse remodelling 6 months after implantation, with reductions in LV end-systolic volume (LVESV) of greater than 15% being the most useful measure (Picard et al., 2012). However, as shown in Table 2, studies examining CRT response use varied definitions, including echocardiographic (LVESV or LVEF), and clinical (NYHA class or clinical composite score). These definitions capture a broad group of patients. The causes of echocardiographic non-response are likely to be completely different from the causes of clinical non-response. For example, whilst sub-optimal LV lead position may lead to echocardiographic non-response, prevalent issues in heart failure such as anaemia, arrhythmia and sub-optimal medical therapy can lead to clinical non-response in the absence of persistent mechanical dyssynchrony (Sieniewicz et al., 2019). As such, the optimal second-line of treatment for these individual patients is also likely to vary, thus providing another cause of the lack of consistency in current studies.

**TABLE 2:** Variations in definition of CRT response across trials.

Study	Definition of CRT response	Intervention
Vijayaraman et al., 2022a	CRT non-response was defined as improvement of LVEF <5% and either worsening or unchanged patient functional status	LBBAP
Brown et al., 2022	Non-responders had an improvement in LV ejection fraction (LVEF) by <5%, and incomplete responders had an improvement in LVEF by >5% with final LVEF <40% at least 3 months post-CRT	CRT optimisation
Naqvi et al., 2006	Symptoms of heart failure post-CRT	CRT optimisation
Sepši et al., 2013	Patients who have developed increase in LVEF >5% and those who had improvement of NYHA class during follow up were classified as responders. Patients who have developed drop in LVEF >5%	CRT optimisation
	and have decreased the NYHA class during the follow up were classified as non-responders. All between were classified as unchanged	
Saba et al., 2022	Non-response defined as unchanged or worsened clinical composite score at 6 months post-CRT.	Multi-site pacing
Leclercq et al., 2019	Response defined as <15% reduction in left ventricular end-systolic volume (LVESV) at 6 months post-CRT.	Multisite pacing
Bordachar et al., 2018	Non-response defined as unchanged or worsened clinical composite score 6 months post-CRT.	Multisite pacing
van Gelder et al., 2016	Non-response defined as remaining NYHA class 3 or 4 at least 6 months post-CRT.	Endocardial pacing
Sidhu et al., 2020	Non-response defines as unchanged or worsening of symptoms or New York Heart Failure (NYHA) functional class after at least 6 months post-CRT.	Leadless endocardial pacing
Chun et al., 2020	Decrease in (LV) end-systolic volume > 15% on echocardiography 6 months after implantation	Sacubitril-Valsartan
Giaimo et al., 2018	Non-response defined as previously treated with CRT for at least 6 months and remained classified as New York Heart Association (NYHA) functional class III or IV despite optimal medical therapy; the echocardiographic assessment showed lack of decrease of the left ventricular end-systolic volume (LVESV) of at least 10% and residual moderate-to-severe or severe FMR.	Mitraclip

Ultimately, determining the optimal second-line pacing intervention in CRT will require well designed clinical trials examining a standardised population of patients, with strict non-response inclusion criteria. Whilst early studies including WiSE-CRT (Sieniewicz et al., 2020), HOT-CRT (Vijayaraman et al., 2019) and LOT-CRT (Jastrzębski et al., 2022) may give us cause for optimism, it is important to avoid extrapolating these results from an unselected population to non-responder groups. For example, no studies have yet shown that the addition of CSP to conventional CRT non-responders will improve outcomes. Indeed, the observational study performed by the LBBAP collaborative study group (Vijayaraman et al., 2022a) suggested that whilst rescue LBBAP was a good alternative treatment for inability to place an epicardial lead via the CS, the response rate of LBBAP in CRT non-responders was poor, with a 30% rate of death or HF hospitalisation within 12 months. It may be that a significant proportion of non-responders are not patients who are receiving inadequate CRT, but rather patients who have an aggressive HF phenotype combined with other co-morbidities, in whom improvement will be challenging to achieve through novel pacing therapies alone. Improvements in outcomes for CRT non-responders have been demonstrated for therapies such as initiation of sacubitril-valsartan (Chun et al., 2020) and transcatheter mitral valve intervention for those with residual moderate/severe mitral regurgitation (Giaimo et al., 2018). These studies highlight the importance of a holistic approach to treating an unwell and high-risk HF population.

In summary, the heterogeneity of the dyssynchronous HF population and the high success rates of empirical conventional CRT mean that generating robust evidence for the optimal pacing alternatives for CRT non-responders is extremely challenging. There is likely a significant subgroup of CRT non-responders who have a superior alternative pacing location, in particular those who have problems with conventional LV lead implantation, or poor LV lead performance due to issues such as high capture thresholds and phrenic nerve stimulation. The plethora of novel therapies including endocardial and conduction system pacing may enable physicians to deliver tailored CRT for individual patients. Further study concentrating on patient selection will ultimately pave the way for this form of precision medicine.
